# Supplementary material for: CD200 Induces Epithelial-to-Mesenchymal Transition in Head and Neck Squamous Cell Carcinoma via β-Catenin-Mediated Nuclear Translocation
Source: Cancers (Basel). 2019 Oct 17;11(10):1583. doi: 10.3390/cancers11101583 (PMC6826410; doi:10.3390/cancers11101583)
Supplement: Supplementary file 1 [file cancers-11-01583-s001.pdf]

Supplementary Materials: CD200 Induces Epithelial-to-Mesenchymal Transition in Head and Neck Squamous Cell Carcinoma via β-catenin-Mediated Nuclear Translocation

Seung-Phil Shin, A-RA Goh, Hyeon-Gu Kang, Seok-Jun Kim, Jong-Kwang Kim, Kyung-Tae Kim, John H Lee, Yong-Soo Bae, Yuh-Seog Jung and Sang-Jin Lee

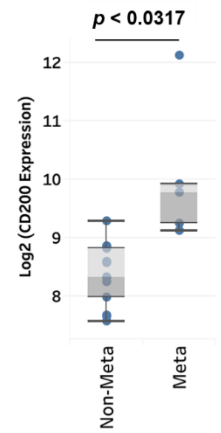

Figure S1. The CD200 expression in metastatic HNSCC patients. CD200 gene expression of metastatic HNSCC is elevated in comparison to that of non-metastatic tumors (two sample *t*-test *p*-value < 0.001) in an independent set of 12 samples on microarrays (GEO accession id, GSE2370). To elucidate a clinical implication of the high CD200 gene expression in head and neck cancer, we downloaded a public dataset of microarray (Affymetrix U95A microarrays) containing 14 non-/metastatic head and neck squamous cell carcinoma from GEO database (accession number: GSE2370). We converted CD200 (probe id: 37716_at) expression level into log2 scale and used two sample *t*-test with a significance level of 0.01 to test for the difference in mean CD200 expression between non- and metastasis groups.

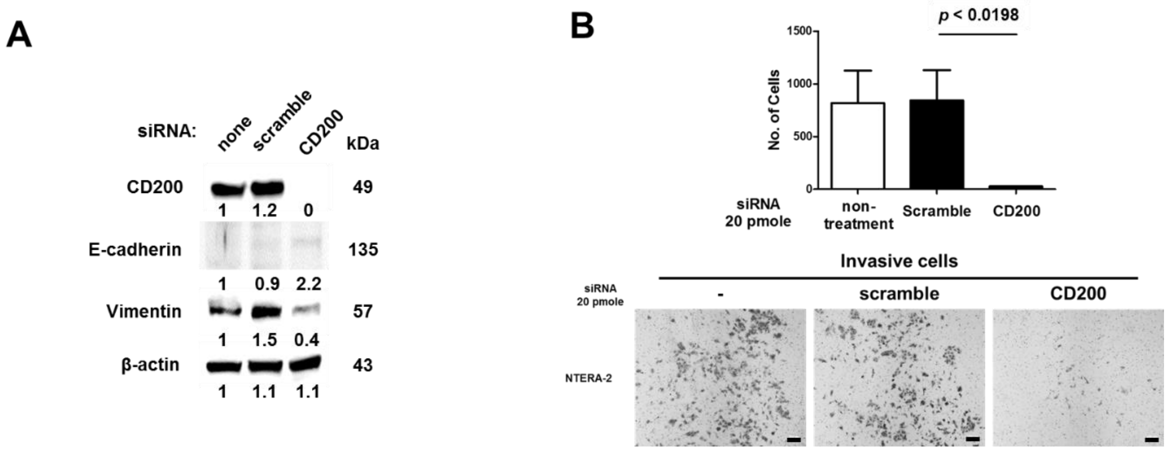

Figure S2. The EMT features in endogenously CD200-expressing NTERA cell. (**A**) The CD200 endogenous expression cell line, NTERA-2, was exposed to 20 pmoles of siRNA (scramble or CD200) for 48 hours. The cells were then digested with RIPA buffer and quantitated. Western blot analysis was performed at 30 µg per sample. The changes of EMT gene by siRNA treatment were detected as anti-CD200, anti-E-cadherin, anti-vimentin and anti-β-actin. (**B**) siRNA treatment for NTERA-2 is the same as supplementary figure 1b. Then, each 1×10^5 cells were added to the matrigel-coated transwell

and incubated for 48 hours. The invaded cells were stained with crystal violet, and then stained cells were directly counted using a microscope (100 μm scale bar). Each error bar in the graph represents the average of three independent experiments (mean ± SEM).

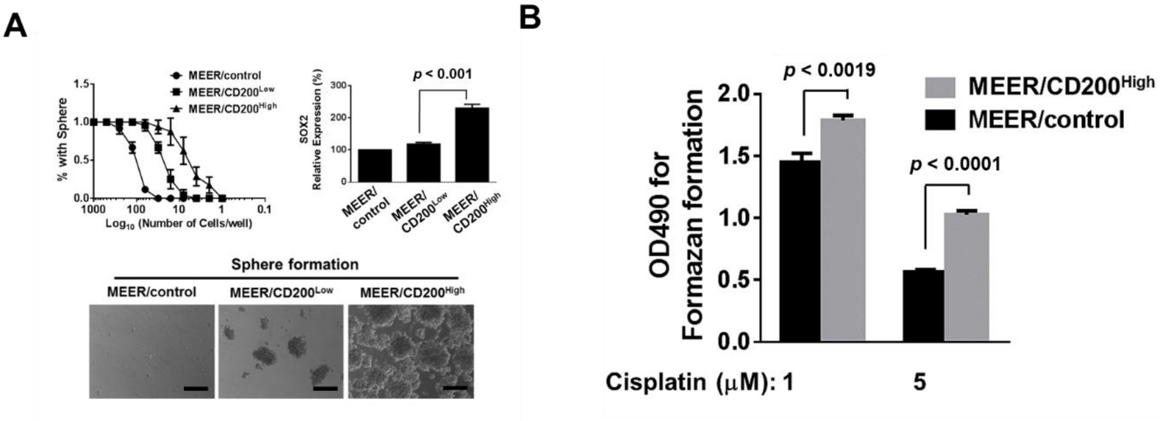

Figure S3. Formation and cisplatin-responsiveness of CD200-overexpressing MEER cell. (**A**) A different number of each cell type was cultured in a suspension media for 7 days. We then counted under a microscope how many spheres were formed and plotted the results (upper left). Total RNA from each cell line was prepared for SOX2 qRT-PCR analysis (mean ± SEM; n = 3). P values were determined by two-tailed paired t-tests (upper right). Cells were cultured in the suspension media and monitored for sphere formation after 7 days (100 μm scale bar, bottom). (**B**) After 1×10^5 cells/well were treated with 1 or 5 μM cisplatin for 48 h, cell viability by MTS assay was determined as a percentage of cells compared to the no-treatment control (mean ± SEM; n = 3) in three independent experiments. Results were analyzed by two-tailed, paired t-tests.

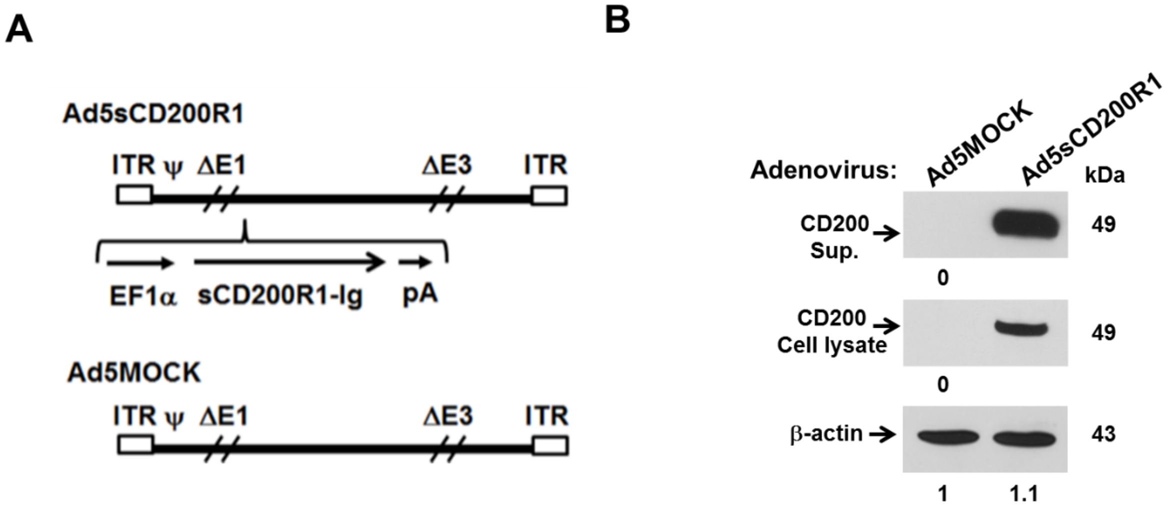

Figure S4. Adenovirus over-expressing soluble extracellular domain of CD200. (**A**) An adenovirus encoding the extracellular domain of CD200R1, sCD200R1, was constructed as a fusion protein containing the Fc portion of mouse IgG2a as described in the Materials and methods. The Ad5MOCK adenovirus was produced as a control. (**B**) HEK293 cells were infected with 10 MOI for 24 h to confirm secretion of soluble sCD200R1-Ig into the culture medium. Culture supernatant (Sup.) was collected, and the cells were lysed with RIPA buffer. Total proteins (40 μg) were resolved for immunoblot analysis.

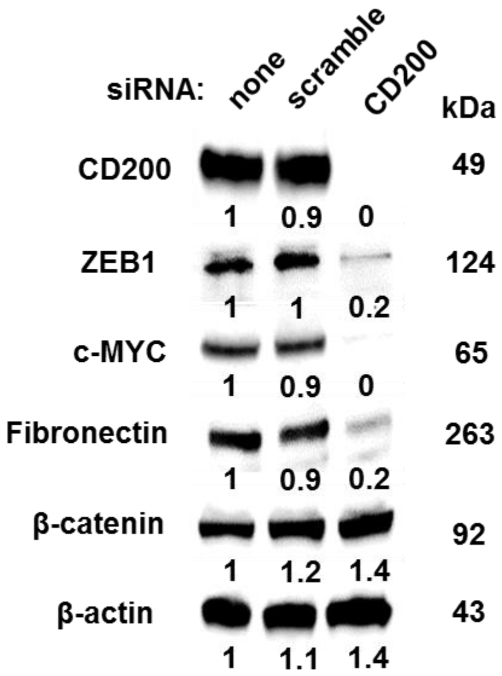

Figure S5. Abolition of β-catenin target genes by CD200 siRNA in CD200 endogenous expression cell. NTERA-2 cells were treated with 20 pmoles siRNA and lysed 48 hours later with RIPA buffer. Western blot analysis was performed at 30 μg per sample. The changes of β-catenin target genes by siRNA treatment were detected as anti-ZEB1, anti-c-MYC and anti-Fibronectin.

Adenovirus construction			sequence
Mouse	CD200R1	Forward	5'- GAA TTC GCC ACC ATG TTT TGC TTT TGG -3'
		Reverse	5'- CAA TGG CTC CTC CTC CTC GTA ATG ATT GGT T-3'
**qRT-PCR**	**EMT gene**		**sequence**
Mouse	CD200	Forward	5'- AAA CAT CCC AGG AAC CCT TG -3'
		Reverse	5'- TGT CTT TGT AGG CAG GCT GG -3'
	N-cadherin	Forward	5'-CTT GAA ATC TGC TGG CTC GC-3'
		Reverse	5'-AGG ATG TGC ACG AAG GAC AG-3'
	E-cadherin	Forward	5'-TGA CGA TGG TGT AGG CGA TG-3'
		Reverse	5'-CAG CCG GTC TTT GAG GGA TT-3'
	vimentin	Forward	5'-AAG CGC ACC TTG TCG ATG TA-3'
		Reverse	5'-TGC TTC AAG ACT CGG TGG AC-3'
	GAPDH	Forward	5'-CCA CCA CCC TGT TGC TGT AG-3'
		Reverse	5'-CCC ACT CTT CCA CCT TCG AT-3'

Figure S6. The primer sequence for adenovirus construction and Quantitative real-time PCR.

Figure 1B

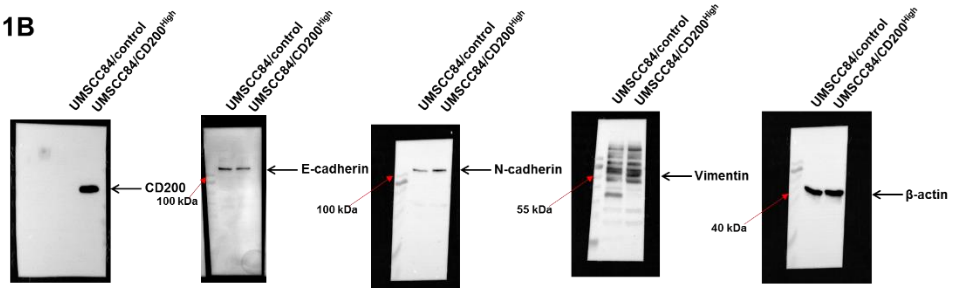

Figure 1C

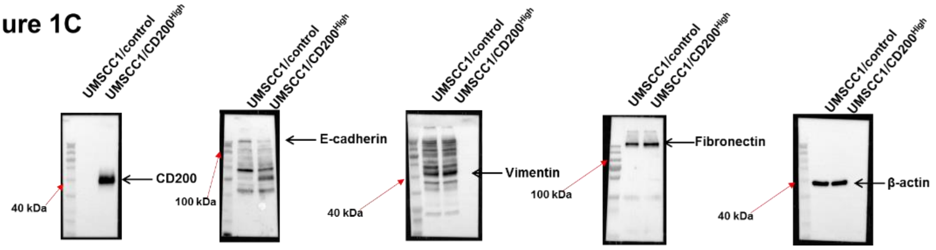

Figure 1D

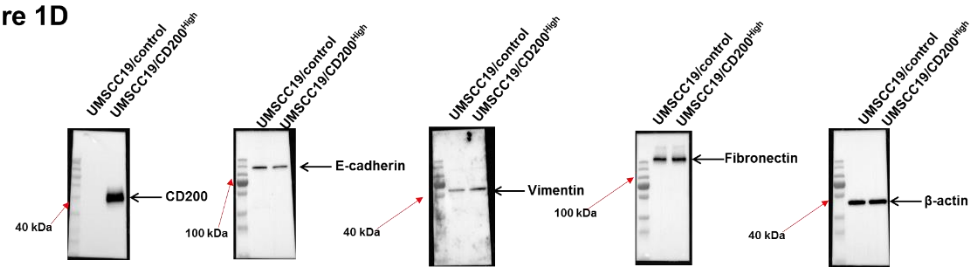

Figure 1E

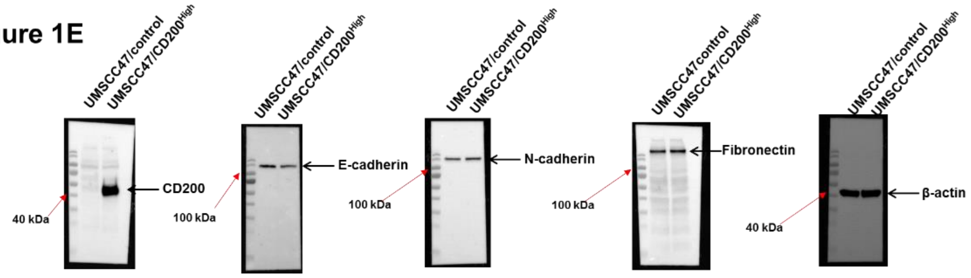

Figure 2C

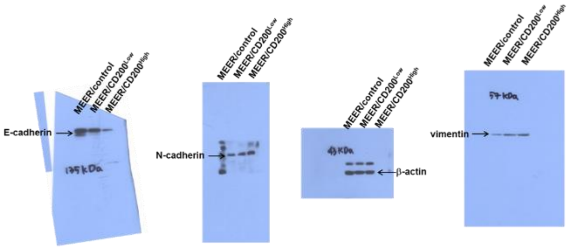

Figure 3A

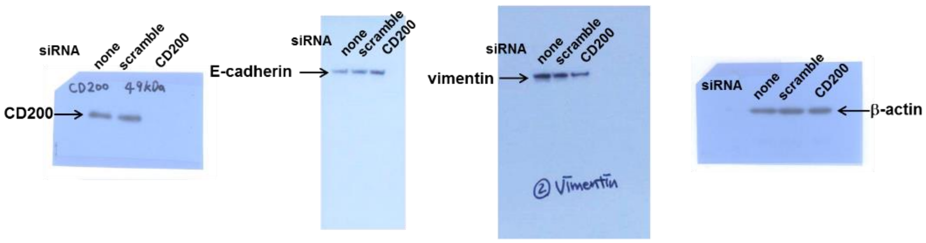

Figure 4A

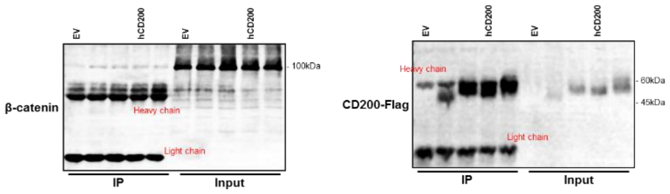

Figure 4B

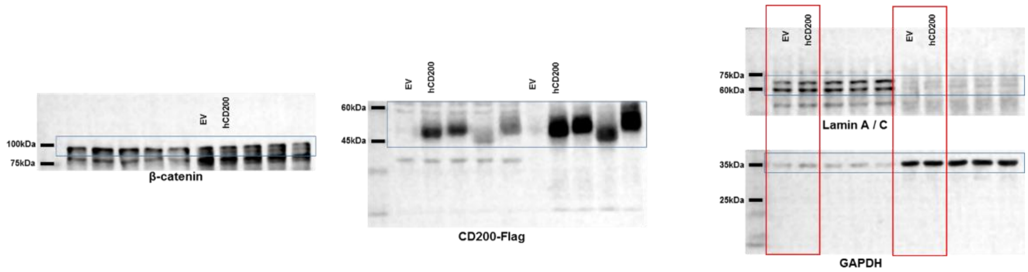

Figure 4E

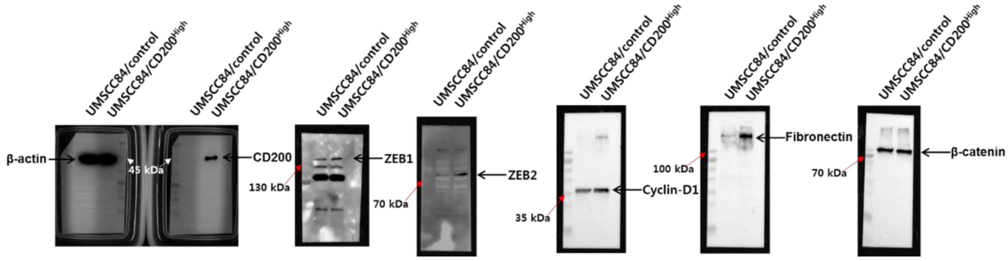

Figure 4F

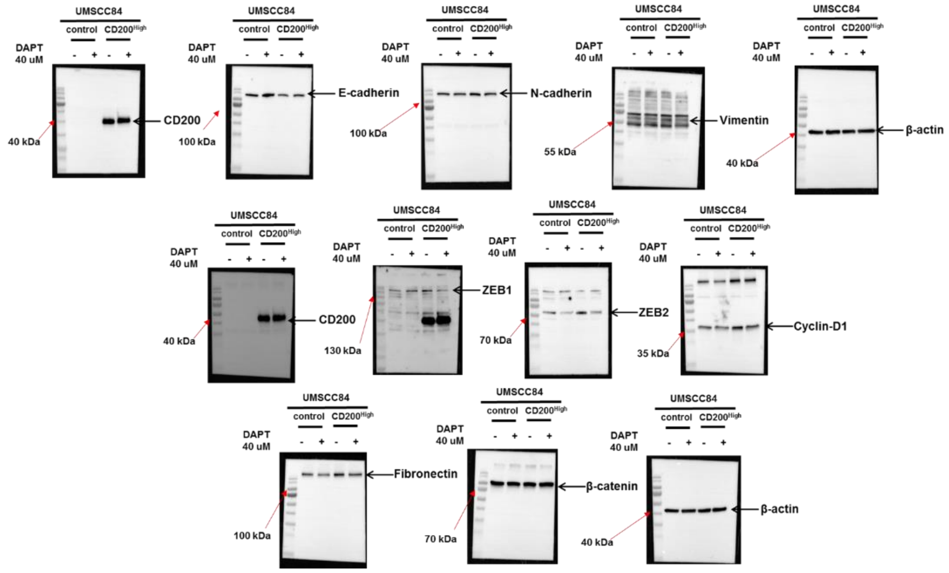

Supplementary Figure 2A

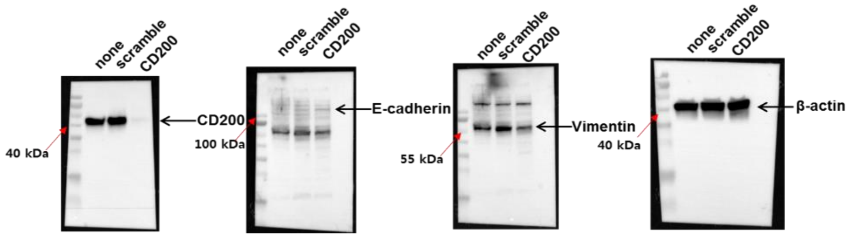

Supplementary Figure 4B

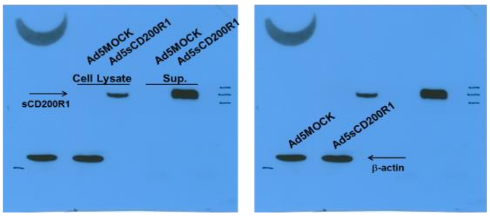

Supplementary Figure 5A

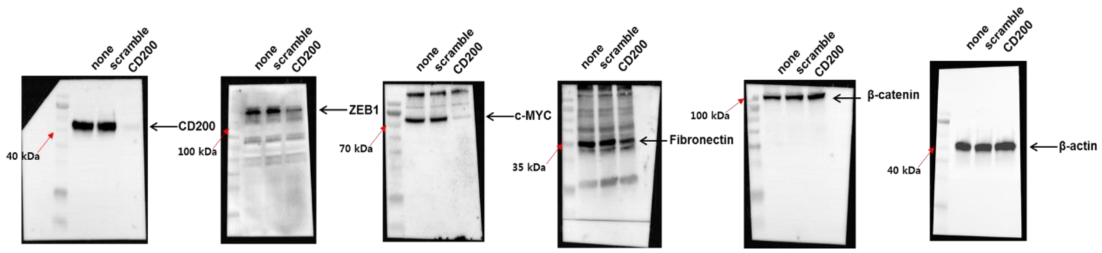

Figure S7. Whole blots showing all the bands of Western blotting presented in Supplementary Figures 1B–E, 2C, 3A, 4A, 4B, 4E, 4F, and Supplementary Figures 2A, 4B, 5A.

© 2019 by the authors. Licensee MDPI, Basel, Switzerland. This article is an open access article distributed under the terms and conditions of the Creative Commons Attribution (CC BY) license (http://creativecommons.org/licenses/by/4.0/).
